# Current Status of Genetic Counselling for Rare Diseases in Spain

**DOI:** 10.3390/diagnostics11122320

**Published:** 2021-12-09

**Authors:** Sara Álvaro-Sánchez, Irene Abreu-Rodríguez, Anna Abulí, Clara Serra-Juhe, Maria del Carmen Garrido-Navas

**Affiliations:** 1 CONGEN, Genetic Counselling Services, C/Albahaca 4, 18006 Granada, Spain; info@congen.es; 2 Genetics Service, Hospital del Mar Research Institute, IMIM, 08003 Barcelona, Spain; abreuire@gmail.com; 3 Department of Clinical and Molecular Genetics, Hospital Vall d’Hebron, 08035 Barcelona, Spain; anna.abuli@gmail.com; 4 Medicine Genetics Group, Vall d’Hebron Research Institute (VHIR), 08035 Barcelona, Spain; 5 U705 CIBERER, Genetics Department, Hospital de la Santa Creu i Sant Pau, Universitat Autònoma de Barcelona, 08193 Barcelona, Spain; clara.seju@gmail.com; 6 Centro de Investigación Biomédica en Red en Enfermedades Raras (CIBERER), 28029 Madrid, Spain; 7 Genetics Department, Faculty of Sciences, Universidad de Granada, 18071 Granada, Spain

**Keywords:** genetic counselling, rare diseases, professional recognition

## Abstract

Genetic Counselling is essential for providing personalised information and support to patients with Rare Diseases (RD). Unlike most other developed countries, Spain does not recognize geneticists or genetic counsellors as healthcare professionals Thus, patients with RD face not only challenges associated with their own disease but also deal with lack of knowledge, uncertainty, and other psychosocial issues arising as a consequence of diagnostic delay. In this review, we highlight the importance of genetic counsellors in the field of RD as well as evaluate the current situation in which rare disease patients receive genetic services in Spain. We describe the main units and strategies at the national level assisting patients with RD and we conclude with a series of future perspectives and unmet needs that Spain should overcome to improve the management of patients with RD.

## 1. The Role of Genetic Counselling for Rare Diseases

Rare Diseases (RD) are defined as such by their low prevalence although their frequency changes depending on the continent. For instance, the European definition of a rare disease is that affecting less than 1/2000 individuals whereas, in the US, this frequency is even lower, affecting only 1/5000 individuals [1,2]. It is worth mentioning at this point the especially challenging cases of ultra-rare disorders, defined as those affecting less than 1/2,000,000 individuals [3]. It is mainly their uncommonness that makes RD so difficult to diagnose producing a diagnostic delay of more than 5 years [4]. Particularly, diagnosis of a rare disease was greater than 10 years in one in five patients in Spain according to a study by the *Spanish* *Federation of Rare Diseases* (FEDER) [5]. Furthermore, complexity of the symptoms, overlap of phenotypes and increasing number of gene/phenotype relationships extremely difficult diagnosis of these patients [6]. To overcome this complexity, different approaches using algorithms and artificial intelligence are being developed [7,8]. It is estimated that around 80% of RD have genetic aetiology [1,2,9,10] implying that genetic testing might be needed at some point of a patient’s lifetime either to effectively reach a genetic diagnosis or to evaluate familial implications, such as recurrence risk of the disorder and identification of other family members at risk [11].

Diagnosis of RD (either clinical, genetic, or both) is not the end of the journey for patients and their families but rather the beginning. Once a genetic diagnosis is achieved, patients need support to understand not only the implications of this verdict and the real meaning of carrying a genetic abnormality but also, and sometimes more importantly, to grief and to adapt to all psychosocial aspects involved [9,12,13]. Furthermore, genetics is shared among the family having implications for the closer relatives accounting for their risk of carrying a disease-causing variant [14]. These disease-causing genetic changes might be passed from one generation to the next, although for rare disorders they also might occur de novo, with the patient being the first affected person in the family [15,16,17]. Inheritance of these variants increases the risk of passing the disease to the next generation, and thus, some relatives who might be carriers will need support, help and guidance to manage and plan a future pregnancy trying to reduce risks of having an affected child [18,19]. However, there are still challenges for the application of cascade genetic testing, such as cost, cultural and social issues, communication (including reduced access to genetic counsellors) and logistic issues (including lack of genetic specialists or the geographical location). For example, living in a different region than the patient testing positive might make family screening of relatives difficult and unequal access to genetics services will also complicate cascade testing [13,14,20]. Furthermore, for some genetic diseases, inheritance patterns and mechanisms of disease are complex and require genetic specialists enriched with communication skills to help families understand the genetic cause and implications for the family [21].

From the patients’ (and their families’) perspective, having a rare disease encompasses a series of milestones that need to be accomplished to reach a final diagnosis and satisfactory disease management. First of all, when a child is born with a rare disease, the roles of the whole family are turned upside down because they now need to cope with a variety of symptoms together with many social, emotional and economical challenges [10,22]. Secondly, the unceasing visits to a long list of specialists not only affects the whole family psychologically and emotionally but also might, in some cases, be detrimental for the diagnosis [23,24]. Since not all specialists work within the same team/setting, clinical information from one specialty might not be correctly (or timely) communicated to others producing a delay in the diagnosis. This is especially important in Spain that is a fragmented country with 17 autonomous regions. Even though economically the Spanish National Health System (SNS) is centralized, access to medical records from different autonomous regions is sometimes challenging, having 15/17 of them access to electronic medical records [25]. Subsequently, parents usually share their situation with other parents and search on the Internet [26] and social media [27] to finally approach some patients with similar clinical characteristics or patient organizations providing support and information [28]. At some point, genetic testing is performed (may be ordered by a clinician with a suspicion of a disease that needs confirmation or as a suggestion from a patient’s advocacy group that shares some knowledge with the parents). More than one genetic testing might be needed to reach a genetic diagnosis and genetic testing will not always be the answer for the parents or the patient [23]; however, there is no doubt that at this point of the journey, patients and their families have already struggled a lot with the healthcare system and received very little (if any) support from it.

Genetic Counselling, either provided by clinical geneticists or genetic counsellors is the communicative process for helping people understand the implications of a genetic variant in their life and health as well as adapting to the medical, psychological and familial consequences of genetic disease [29,30]. The role of a genetic counsellor involves many tasks (see Supplementary Table S1 for core competencies) that are expected to accomplish in order to ensure an individual has enough personalized information and support to be able to deal not only with their genetic disease as well as to make informed decisions [31,32,33]. Ideally, from the very first visit to a specialist, patients should receive support from a genetic counsellor (in coordination with multidisciplinary genetics units) being able to dig around the clinical and family data and offer an adequate clinical letter to patients. This might help identification of potential inheritance patterns, but more importantly, will assist families to cope with difficulties along the way by empowering them with knowledge and referring them to specialists (neurologist, ophthalmologist, nephrologist, social services, etc.) for their follow-up [12,34]. In fact, a study evaluating the economic impact of using genomic sequencing to diagnose RD highlighted that parents find genetic counsellors to be a valuable resource facilitating complex decisions [11] and some others highlight the cost-effectiveness of providing Genetic Counselling [35,36]. Finally, it is worth mentioning the harm produced when no appropriate Genetic Counselling is provided to RD patients, impacting at many levels [37]. This negative outcome might be traduced into psychosocial effects, inappropriate genetic tests, misinterpretation of results, or even inadequate disease management that consequently bring distress and discomfort to patients [38]. Despite all of this, the state of this profession globally varies widely among continents and even countries [39].

Unfortunately, the situation is particularly difficult in Spain which is, at the moment, the only European country not legally recognizing clinical geneticists as healthcare professionals and thus impeding patients to have access to these specialists [30]. Reaching legal recognition will have a profound impact not only on clinical geneticists (as they will be considered part of the healthcare community, facilitating access to promotion, specific training, equality on the salary and conditions, etc.) but also will tremendously impact patients. Once clinical geneticists are recognized as part of the healthcare team, referral to those specialists will be easier, faster, and more efficient so patients with RD will benefit from receiving more integrative clinical supervision.

Due to this circumstance, each hospital delivers genetic services in a unique way, existing as highly specialized centres or others with scarce or even absence of this service. The main objective of this paper was to evaluate the current state of Genetic Counselling in Spain particularly focusing on the management of RD. To do so, we will describe the main units and strategies at the national level aiding patients with RD and we will highlight the main future trends and unmet needs that Spain needs to overcome to improve the management of patients with RD.

## 2. Genetic Services (Including Genetic Counselling) for Rare Diseases in Spain

**Figure 1:**
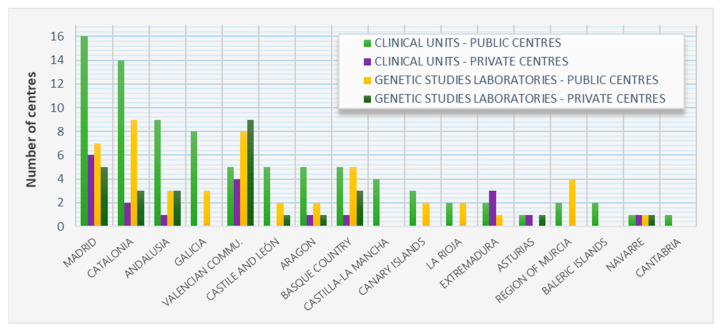
Number of public and private centres in Spain providing Genetic Counselling services by region. Source: Spanish Society of Medical Oncology (SEOM), 2021.

Despite the lack of recognition in Spain of Clinical Genetics as a healthcare specialty, the current legislation for biomedical research states that any specialist with sufficient genetics background and communication skills can provide Genetic Counselling [40]. According to the current records, specific Spanish hospitals provided Genetic Counselling since the 1970s. As recommended by the European Society for Medical Oncology (ESMO) and the American Society of Clinical Oncology (ASCO), an oncologist should be able to identify inheritance patterns predisposing to hereditary cancers [41] and this statement has driven an improper use of Genetic Counselling in oncological settings. Thus, historically, Genetic Counselling Units in Spain primarily were associated with Cancer Genetic Counselling. In fact, the Spanish Society of Medical Oncology (SEOM) has in its website (https://seom.org/informacion-sobre-el-cancer/consejo-genetico/unidades-consejo-genetico (accessed on 5 December 2021) an updated list of specialized centres providing Genetic Counselling in the country (most of them providing Genetic Counselling in the oncological field but also in other areas, such as reproduction, neurology or cardiology among others). According to the most updated version, Spain counts with 85 public and 20 private units which include genetic counselling among their services, as well as 49 public and 27 private laboratories dedicated mainly to performing genetic studies but most of which have specialists offering Genetic Counselling (in this case mainly focussed to the specialists ordering the tests) (Figure 1). However, in most cases, professionals providing Genetic Counselling do not have specific training. In fact, it is important to highlight that there are only 29 European registered genetic counsellors in Spain, which contrasts with the number of genetic services. The realization that genetics has a strong impact not only on preventing but also treating cancer has led to the implementation of genetic studies, such as multi-gene panels [42] to identify high-risk individuals allowing family screening studies. Furthermore, the increasing use of genetic variants identification (either in the tumour itself or at the circulating level) for targeted treatments in cancer [2,43] supports the implementation of Genetic Counselling in this area. However, no such effort has been made regarding other genetic diseases, such as RD to clearly assess the advantages of implementing Genetic Counselling to improve current care provided to affected patients and their families in Spain.

Another clinical area being historically related to Genetic Counselling with a special interest for RD is reproductive medicine. According to the *European Society of Human Genetics* (ESHG) and the *European Society of Human Reproduction and Embryology* (ESHRE), Genetic Counselling should always be provided to any patient showing infertility as well as for prenatal genetic diagnosis (PND) [44]. In fact, genetic services in the area of reproductive medicine in Spain have been largely provided by gynaecologists and they ordered genetic tests after positive prenatal screening or upon advanced maternal age to confirm the presence of chromosomal anomalies [45] without the inclusion of a genetic specialist in the process. With the implementation of carrier screening tests [19], a gynaecologist can also evaluate the risk of a couple (or even gamete donors) of carrying genetic variants responsible for autosomal recessive or an X-linked genetic disease. Thus, they might be able to reduce the risks of having an affected child with diseases, such as cystic fibrosis [46] or X-fragile by the use of preimplantation genetic diagnosis (PGD) [47]. Also, prenatal genetic testing (either non-invasive as a screening method or invasive as a diagnostic tool) might allow early detection of genetic abnormalities. Finally, in neonatal care, newborn screening can also reduce diagnosis time for a genetic disease allowing rapid actions to be taken [18,24]. Thus, as methodology and genetic information become more complex and options in the reproductive field are increasing, other professionals, such as biologists, embryologists, clinical geneticists, genetic counsellors, and clinical laboratory geneticists need also to be involved from a clinical care perspective. For example, regarding foetal anomalies that eventually led to pregnancy termination, a recent study evaluated the importance of providing personalized counselling and support facilitating the grieving process [48] and in this case, it was a duty of obstetric-gynaecologic nurses, mainly.

Regardless of the efforts described above and even though Genetic Counselling is well defined in the Spanish law for Biomedical Research [40], recognition of clinical geneticists, genetic counsellors and clinical laboratory geneticists as healthcare specialists is still an unsatisfied demand. Although with different competencies for each specialty (see Supplementary Table S2), they all perform complementary tasks that eventually improve patient management. Whereas one professional profile is focused on clinical diagnosis (clinical geneticist), another is centred on emotional support to the patient (genetic counsellor) and the third one is responsible for providing adequate tools to perform genetic testing (laboratory geneticist). Several attempts tried to develop a certification for the training of these professionals although there is not a national strategy that establishes a roadmap for this educational path. For example, the Spanish Association for Human Genetics (AEGH) developed an accreditation system open to graduates in medicine, biology, biochemistry, pharmacy and chemistry [49]. Regarding Master’s education, the University Pompeu Fabra successfully achieved four graduations for a master’s degree training future genetic counsellors. Students from the first promotion of this Master founded the Spanish Association of Genetic Counselling (SEAGen) creating awareness about the need for professionalization. More recently (2019–2021) the Autonomous University of Barcelona also promoted an MSc in Healthcare Genetics with three paths: clinical genetics, clinical laboratory genetics and Genetic Counselling. Currently, the only itinerary accredited by the European Board of Medical Genetics (EBMG) is the one for Genetic Counselling. However, none of these efforts have been legally recognized as official training and professionals specialized in Medical Genetics (Clinical Geneticists, Genetic Counsellors and Clinical laboratory Geneticists) still have difficult access to work as healthcare specialists in Spain [30,34], with the detriment that this causes to patients.

**Figure 2:**
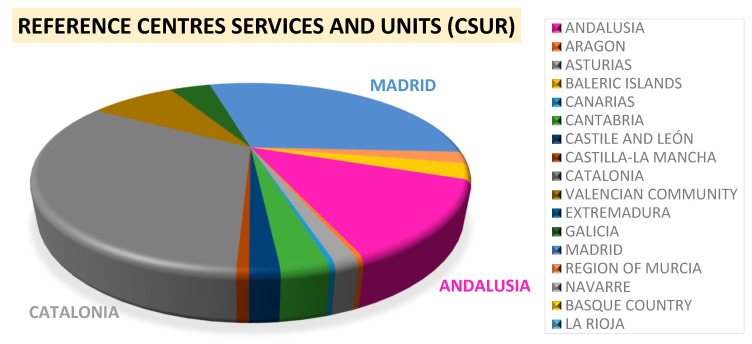
CSUR distribution. Most of these *Reference Centres*, *Services* and *Units* (CSUR) are in the regions of Catalonia, Madrid, and Andalusia. Source: Listado CSUR, 2021.

In contrast with the lack of specific Genetic Counselling Services for RD in Spain, highly specialized multidisciplinary units and/or centres (CSUR, *Reference Centres, Services and Units*) for particular diseases (some of them of low prevalence) are being created since 2008. Currently, there are 71 specific disease groups that are covered by up to 297 CSUR in the country. However, one of the main objectives of these units is not necessarily medical assistance but to become a reference contact for disease management strategies definition as well as support for both, patients and clinicians dealing with a particular disease [50]. In fact, these centres or units belong to public hospitals that agglutinate a series of clinical specialists who are experts in specific disease groups (but they are not a genetic service itself). Clinicians join creating multidisciplinary groups to serve as reference centres for the whole country, but as genetic counsellors are not usually incorporated in these groups, patients lack this service. Clinicians in these CSUR might be specialized in specific methodologies and are able to define therapeutic strategies or function as consultants for the general practitioner of the patients with these pathologies but do not necessarily deal with the patient directly offering genetic counselling. Regarding these CSUR, there are three main regions in Spain accumulating most of these centres: Catalonia (97/297), Madrid (89/297) and Andalusia (39/297) (Figure 2), implying that most patients need to move from their region to a different one to receive specialized disease management. Despite being specialized in some genetic diseases, this CSUR does not provide Genetic Counselling as a service, neither do other centres offering genetic testing to their patients.

**Figure 3:**
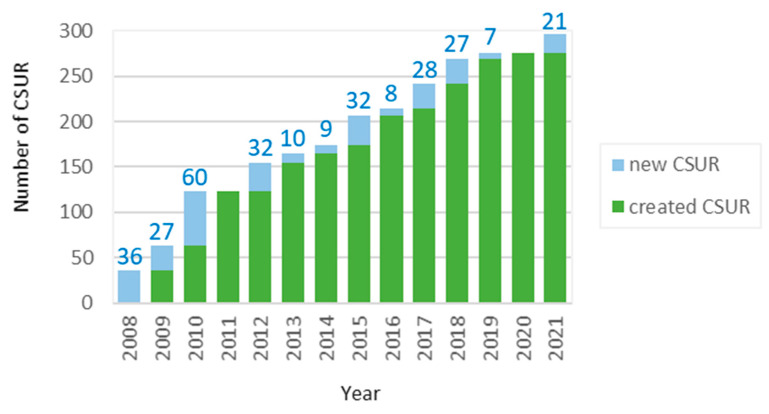
Number of *Reference Centres*, *Services* and *Units* (CSUR) along the time. Source: Listado CSUR, 2021.

Since the establishment of the first 36 CSUR in 2008, there has been a great expansion of these centres, reaching a total of 297 in 2021 (Figure 3). In addition, as new CSURs are established, the range of RD to which they give support is rising (see list of https://www.mscbs.gob.es/profesionales/CentrosDeReferencia/docs/ListaCSUR.pdf) CSURs (accessed on 5 December 2021). Of the 71 groups of diseases, the most prevalent are those treating childhood eye disorders, congenital and/or family heart diseases, metabolic and neuromuscular diseases, neurocutaneous disorders, epidermolysis bullosa, rare hereditary anaemias and congenital coagulopathies, among others. These CSUR allow continuous patient care independently of their age. Currently, there are 74 paediatric CSUR, 109 specialized in adult diseases and 114 attending both children and adults.

Contrarily to the difficulties encountered for the recognition of the profession that impedes in many cases access to these specialists, Spain has made a significant effort for several years to create national strategies and documents supporting patients with RD.

One example is a compendium created in conjunction with the *Institute for Research of Rare Diseases* (IIER) and the *Institute of Health Carlos III* (ISCIII) summarizing the main RD by organs and giving a brief introduction about orphan drugs, social services and other aspects of interest [51]. Also, since 2008 collaboration between National Alliances and EURORDIS implemented the National Plans and Strategies for RD with several joint actions to integrate policy measures [3,52]. In 2011 the ISCIII and the *Rare Diseases Network Biomedical Research Consortium* (CIBERER) created a national registry for patients with RD to be used in research [53]. In fact, lots of investments and actions were made to promote research in the RD field, neglecting improvements in the clinical, daily life of patients that need continuous support and advice regarding their disease. Collaborations between research centres and hospitals allowed concentrating efforts in particular diseases, such as neuromuscular [54,55], retinal [56,57] and cardiovascular [58] rare diseases among others, to provide the most comprehensive management, including Genetic Counselling, although this effort should be extended to ideally all RD.

## 3. Current Needs and Future Directions of Genetic Counselling for Rare Diseases

The so-called “diagnostic odyssey” is the process during which most patients with a rare disease and their families try to find the cause, name, prognostic and treatment of their disease [20,59]. This long journey usually begins with the first symptoms and hopefully (but not always) ends a few years later, with the diagnosis of the disease [9]. The future of RD envisions a faster diagnosis thanks to high-throughput Next Generation Sequencing (NGS) technologies as well as artificial intelligence [60] with the intention of helping clinicians to shorten the time to diagnosis. Several studies demonstrate the utility of whole-exome (WES) [61,62] or whole-genome (WGS) [63,64] sequencing, as well as including other omics, such as RNA-seq or methylation profiles, increasing the diagnostic yield particularly on undiagnosed individuals with a suspected genetic condition [65,66]. Nevertheless, the improvement in diagnostic efficiency comes with several shortcomings, such as cost and time/analysis load [24,67] that are expected to be solved in the near future as technology improves in efficiency. However, it is important to recognise diagnostic limitations for each technology and it is at this point at which genetic (or genomic) counsellors provide an added value. In addition, there are other limitations from the ethical point of view that might have a stronger impact on patients and are not so easy to tackle. Some of these are identification and interpretation of variants of uncertain significance (VUS), secondary and incidental findings, or equal access to genetic information, among others [66,68,69]. Furthermore, the fact that some patients have multiple disease-causing variants (polygenic diseases) and also several genetic diagnoses complicate the interpretation of genetic data, and thus, require expert geneticists (all three specialties: clinical geneticists, genetic counsellors and clinical laboratory geneticists) to provide enough information and support to patients [34,70].

Following the recommendations of the American College of Medical Genetics (ACMG) and the European Board of Medical Genetics (EBMG), three Spanish scientific societies (AEGH, SEQC-ML and SEOM) elaborated a consensus document for the implementation of NGS multigene panels in the evaluation of hereditary cancer predisposition in which Genetic Counselling takes place both before and after genetic testing [71]. However, no such document is produced specifically for RD, and thus, not all patients undergoing a genetic test for diagnostic purposes receive a Genetic Counselling pre-test and/or post-test session. In fact, this service should be of greater importance when accounting for WES/WGS as the possibility of identifying secondary findings and/or VUS is greater [65,66,72,73].

Shortening the diagnostic process and providing adequate management to patients with a rare disease in Spain necessarily implies a series of changes both in the SNS and in the Spanish society. The 2014 update of the Strategy for Rare Diseases of the Spanish National Health System proposed a series of Action Strategies for the prevention and early detection of RD [74]. One of them claims that patients should have better access to diagnostic tests and Genetic Counselling services. In fact, most patients in Spain are unable to reach an early diagnosis mainly due to inadequate approach as a consequence of a deficiency in the training of clinicians as specialized training for Clinical Genetics does not exist [5,29]. This, together with the lack of recognition of genetic counsellors to support patients, is translated into inappropriate genetic tests lengthening the diagnostic process, increasing clinical expenses, and adding anxiety to patients. Another of the needs highlighted in this strategy is to provide continuous training to specialists who make up these multidisciplinary teams, with emphasis on Primary Care professionals. Particularly, in the context of Primary Care, an online tool (DICE-APER protocol) was developed to speed up the diagnosis of RD [74,75]. However, many patients refer to the lack of knowledge about RD by the general practitioner taking care of them [76]. This situation is shown in the studies by Ramalle-Gómara et al. and Bueno et al. in which even clinicians are aware of the lack of knowledge and specific training they have regarding RD [59,77]. In addition, adequate training of these specialists will improve the emotional state and well-being of patients and their families, as many perceive a feeling of abandonment that forces them to turn to patient organizations for support [78].

## 4. Conclusions

The main goal of this paper was to provide a comprehensive overview of the current situation of Genetic Counselling in Spain, particularly focussed on RD. To introduce the matter, we started by describing the importance of providing Genetic Counselling to patients with RD. Then, we continued by describing the main genetic services providing Genetic Counselling in Spain with special interest to those highly specialized for RD. We also made a brief description of some of the research initiatives in which Spain is involved regarding RD and we finished by highlighting some of the current needs and future directions in Genetic Counselling for RD.

We emphasised that the current situation of Genetic Counselling for RD in Spain is jeopardised by the lack of recognition of Clinical Genetics as a healthcare specialty within the SNS. This must include three professional profiles: clinical genetics, genetic counselling, and clinical laboratory genetics. Currently, there are more than 3 million people in Spain affected by a rare disease and the implementation of next generation sequencing technologies is improving diagnostic yield. By understanding this reality, we ought to take advantage of these technologies to improve patient management. However, it is necessary to solve the lack of access to specialized professionals who are capable of analysing and interpreting this information. Thus, it is necessary to create multidisciplinary teams providing integrative solutions for the needs of patients with RD. Ideally, these clinical genetics services would include a team of specialist physicians, nurses, psychologists, as well as new professional profiles, such as bioinformatics.

Clinical guidelines and management strategies must be implemented at the national level allowing better coordination between different centres to make society/professionals aware of the existence of CSURs and to create new ones capable of correctly managing patients regardless of their residence location. Furthermore, the incorporation of specialists providing Genetic Counselling into multidisciplinary groups dealing with RD patients is essential to provide personal and familial information and support accompanying them throughout this uncertain and unpredictable journey. Furthermore, the actualization of the current registries at the national level to estimate the prevalence of RD, to understand their distribution is needed to develop appropriate action strategies.

Finally, updated and continuous training of health professionals on the new advances for diagnosis and treatment of RD will be necessary to guarantee equal access of patients to quality health services, thus improving the management of their disease and consequently, improving their quality of life.

## Data Availability

Data from this review was extracted from the following links: https://seom.org/informacion-sobre-el-cancer/consejo-genetico/unidades-consejo-genetico (accessed on 5 December 2021); https://www.mscbs.gob.es/profesionales/CentrosDeReferencia/docs/ListaCSUR.pdf (accessed on 5 December 2021).

## Supplementary Materials

The following are available online at https://www.mdpi.com/article/10.3390/diagnostics11122320/s1. Table S1. Core competences of Genetic Counsellors adapted from the European Board of Medical Genetics (EBMG) recommendations; Table S2. Summary of the main tasks performed by the different healthcare professionals that should be involved in RD patients’ management.
