# Supplementary material for: Current Status of Genetic Counselling for Rare Diseases in Spain
Source: Diagnostics (Basel). 2021 Dec 9;11(12):2320. doi: 10.3390/diagnostics11122320 (PMC8700506; doi:10.3390/diagnostics11122320)
Supplement: Supplementary file 1 [file diagnostics-11-02320-s001.zip › diagnostics-1480703-supplementary.pdf]

Supplementary Table S1. Core competences of Genetic Counsellors adapted from the European Board of Medical Genetics (EBMG) recommendations.

	Competence	Tasks
COUNSELLOR-CLIENT RELATIONSHIP	1. Establish relationship and clarify clients' concerns and expectations.	1.1 Creating environment for expression of feelings, anxieties, beliefs, and expectations considering clients' experiences.<br>1.2 Identifying client needs.<br>1.3 Enabling clients to make informed choices about the implications of their family history.<br>1.4 Taking appropriate action to meet identified needs with the agreement of the client.
	2. Make appropriate and accurate genetic risk assessment.	2.1 Identifying sufficient medical, family and personal information to make appropriate genetic risk assessment.<br>2.2 Ascertaining medical information from other sources to confirm family information and diagnosis.<br>2.3 Understanding inheritance patterns and the underlying mechanisms by which genetic disease may occur.
	3. a. Convey clinical and genetic information to clients<br>3. b. Explain options available including risks, benefits and limitations.<br>3. c. Evaluate the understanding of the individual related to the topics being discussed.<br>3. d. Acknowledge the implications of individual and family experiences, beliefs, values and culture.	3.1 Providing information about the genetic disorder appropriate to the client's needs, reflecting their values, religious and cultural beliefs and preferences.<br>3.2 Providing information based upon appropriate interpretation of genetic and clinical knowledge.<br>3.3. Communicating with respect to the genetic risk assessment and possible options.<br>3.4 Supporting dissemination of information about the genetic disorder to at risk relatives by the client.
	4. Make an assessment of clients' needs and resources and provide support, ensuring referral to other agencies as appropriate.	4.1 Ascertaining psychological needs of the individual or family.<br>4.2 Respecting clients' preferences, providing support and making referrals to other agencies.<br>4.3 Identifying and supporting clients' access to local, regional and national resources and services.
	5. Use of a range of counselling skills to facilitate clients' adjustment and decision-making.	5.1 Using safe, effective and appropriate counselling skills to support clients to make adjustments and decisions.
MANAGEMENT AND ORGANIZATION	6. Document information including case notes and correspondence in an appropriate manner.	6.1 Systematical collection and maintenance of comprehensive and accurate records detailing any intervention.<br>6.2 Maintaining confidentiality and security of written and verbal information.
	7. Find and utilize relevant medical and genetic information for use in Genetic Counselling.	7.1 Collecting, evaluating and using relevant information about the genetic disorder in question.<br>7.2 Critically assessing current evidence to inform practice and professional development.<br>7.3 Disseminating evidence of good practice and service improvement through verbal and written media.
	8. Demonstrate ability to organize and prioritize a case load.	8.1 Addressing client needs in a sensitive and fair manner making best use of resources available.<br>8.2 Prioritizing according to patient needs.
	9. Plan, organize and deliver professional and public education	9.1 Explaining of how genetics impacts on affected individuals, their families, partners and carers.<br>9.2 Seeking to raise awareness of available services and resources related to genetic healthcare.<br>9.3 Acting as a resource for other professionals and lay groups.
PROFESSIONAL PRACTICE AND ETHICS	10. Establish effective working relationships to function within a multi-disciplinary team and as part of the wider health and social care network.	10.1 Promoting patient-centered care in partnership with the client, their family, and appropriate care providers.<br>10.2 Facilitating communication via a strong multidisciplinary network of professional and lay colleagues.<br>10.3 Coordinating patient and family care as appropriate.
	11. Contribute to the development and organization of genetic services.	11.1 Evaluating own practice and that of others in the light of new evidence and modifying practice if necessary.<br>11.2 Using critical skills to consider how new evidence may improve service organization and delivery.

		11.3 Actively seeking opportunities to meet with colleagues to discuss professional issues and innovations.<br>11.4 Actively seeking opportunities to collaborate with colleagues in audit and research to improve client care.
	12. Practice in accordance with an appropriate code of ethical conduct.	12.1 Encouraging professional standards of safe and ethical practice at all times.<br>12.2 Using professional standards of practice to evaluate own and others' performance.<br>12.3 Recognizes the duty to seek professional advice if standards of care are threatened.<br>12.4 Contributes to the debate on ethical challenges in genetic practice. 12.5 In normal circumstances discloses information about individuals to appropriate third parties only with the client's permission.
	13. Recognize and maintain professional boundaries and limitations of own practice.	13.1 Recognizing practice limitations and demonstrating referrals to other health professionals when appropriate.<br>13.2 Consulting other health professionals when the client's needs fall outside the scope of genetic practice.<br>13.3 Referring clients to colleagues when necessary.
PROFESSIONAL DEVELOPMENT	14. Demonstrate reflective skills and personal awareness for the safety of individuals and families.	14.1 Demonstrating reflective practice, which informs future clinical interactions.<br>14.2 Maintaining a portfolio recording reflection on practice.<br>14.3 Accessing counselling and/or clinical supervision to underpin and enhance practice.
	15. Present opportunities for clients to participate in research projects in a manner that facilitates informed choice.	15.1 Enables clients to make an informed choice on whether to participate in a research project or not.
	16. Demonstrate continuing professional development as an individual practitioner and for the development of the profession.	16.1 Actively seeks opportunities to update knowledge and skills, and reflects on the implications of these for own practice and that of professional colleagues.

Supplementary Table S2. Summary of the main tasks performed by the different healthcare professionals that should be involved in RD patients' management. A green tick indicates a task that is usually carried out by one professional profile and a red cross, a task that should not be performed by a particular professional profile.

Tasks	Genetic Counsellor	Clinical Geneticist	Laboratory Geneticist
**Diagnosis of genetic diseases**	✗	✓	✓
Clinical diagnosis	✗	✓	✗
Laboratory diagnosis	✗	✗	✓
**Disease management**	✓	✓	✗
Clinical management	✗	✓	✗
Therapeutic management	✓	✓	✗
Psychological management	✓	✗	✗
**Risk assessment**	✓	✓	✗
Identify relatives at risk	✓	✓	✗
Help understanding risks	✓	✗	✗
**Genetic testing**	✓	✓	✓
Help understanding if necessary	✓	✗	✗
Help selecting the most appropriate test	✓	✗	✓
**Interpreting genetic results**	✓	✗	✓
Providing tools to patients to manage results	✓	✗	✗
**Counselling**	✓	✗	✗
Empowering patients to take informed decisions	✓	✗	✗
Emotional support	✓	✗	✗
